# Determination of melatonin by a whole cell bioassay in fermented beverages

**DOI:** 10.1038/s41598-019-45645-7

**Published:** 2019-06-24

**Authors:** María Ángeles Morcillo-Parra, Gemma Beltran, Albert Mas, María-Jesús Torija

**Affiliations:** Departament de Bioquimica i Biotecnologia, Facultat d’Enologia, Universitat Rovira i Virgili, Tarragona, Spain

**Keywords:** Assay systems, Biochemical assays

## Abstract

Melatonin is a bioactive compound that is present in fermented beverages, such as wine and beer, at concentrations ranging from picograms to nanograms per mL of product. The purpose of this study was to optimize a novel fluorescent bioassay for detecting melatonin based on a cell line that contains the human melatonin receptor 1B gene and to compare these results with LC-MS/MS as a reference method. Conditions that could affect cell growth and detection (cell number per well, stimulation time, presence or absence of fetal bovine serum and adhesion of cells) were tested in the TANGO^®^ cell line. Food matrices (wine and grape must) could not be directly used for the cell line due to low response. Therefore, for the determination of melatonin in food samples, an extraction procedure was required before conducting the assay. We demonstrated an improvement in melatonin determination by the cell-based bioassay due to increased sensitivity and specificity and improved quantification in complex matrices. Therefore, this method is a good alternative to determine melatonin content in some food samples, especially for those containing very low melatonin levels.

## Introduction

Melatonin (N-acetyl-5-methoxytryptamine) (Mel) is an indoleamine that has been identified within a wide range of invertebrates, plants, bacteria and fungi^1,2^. Therefore, Mel is considered a ubiquitous molecule that is present in most living organisms^2^.

Mel is a bioactive molecule that participates in many physiological processes in the human body, including the regulation of the circadian rhythm^3^ and as an antioxidant via receptor-independent processes^4,5^. Recently, Mel has also been associated with a protective function against oxidative stress and UV radiation in yeast^6,7^.

Mel has been found in many fermented beverages, such as beer or wine, at concentrations ranging from picograms to nanograms per mL of product^8–10^. In fact, during the wine-making process, the concentration of Mel reaches its maximum between the first and second day of fermentation^11,12^, highlighting the role of yeast in its production. Despite the occurrence of Mel in fermented beverages is low, these concentrations have been described as contributing sufficiently to the dietetic intake to exhibit measurable effects^13^.

Mel can be determined by high-performance liquid chromatography (LC) with fluorescence (FL)^14–16^, mass spectrometry (MS)^17–19^, gas chromatography and mass spectrometry (GC-MS)^20^, radioimmunoassay (RIA)^20^, enzyme-linked immunosorbent assay (ELISA)^18,21,22^ and immunoprecipitation^23^. Because Mel production by yeast is very low^11,17^, these methods have limited sensitivity or are technically complex to be adapted as a routine technique for the rapid detection of the presence of Mel in yeast-derived samples. For this reason, it is important to develop new methods for Mel determination in fermented beverages.

In this context, mammalian cell-based assays have been used with numerous reporter genes for monitoring gene or enzyme activity^24^, such as luciferase, β-galactosidase enzyme (*lacZ*) or green fluorescence protein (GFP). Nonetheless, these reporter technologies have several shortcomings as follows: bioluminescence of D-luciferin is transient, the *lac*Z system requires membrane permeabilization and despite the main advantages of GFP (non-invasive and no substrate requirement), its detection limit is very high because there is no enzyme amplification^25^.

The β-lactamase enzyme (BLA) is another commonly used reporter system for gene expression and enzyme activity. Nevertheless, the effectiveness of the BLA system as a reporter gene was not used until Zlokarnik *et al*.^26^. reported the synthesis of a fluorogenic BLA substrate, CCF2/4. CCF2/4 is composed of two fluorescent dyes, 7-hydroxycoumarin-3-carboxamide and fluorescein, bridged by cephalosporin. With the CCF2/4 substrate, as few as 50 molecules of BLA can be detected within a cell^26^. The BLA system also provides high sensitivity due to the absence of endogenous BLA activity in mammalian cells. BLA system could be used with several receptors (such as a Mel receptor, MTNR1-B), where fluorescence detection is caused by fluorescence resonance energy transfer (FRET) system. When the BLA substrate is intact produces green fluorescence, whereas when the fluorophores are separated, the FRET fluorescence is lost and fluorescein generates blue fluorescence. Therefore, when there is no Mel (Fig S1a), the BLA system is not activated and the CCF2/4 remains intact, producing green fluorescence. In contrast, when there is Mel in the medium (Fig S1b), it is recognized by the MTNR1-B receptor, which activates the BLA reporter gene through a transcriptional factor. BLA enzyme cleaves CCF2/4, generating blue fluorescence. The relation between blue and green fluorescence allows quantifying Mel, by using a standard calibration curve.

BLA-based assays are useful for studying signalling pathways in live cells^27^, gene families including GPCRs^28^, protein folding^29^, adaptive and targeted gene evolution^30,31^, protease activity^32^, gene trapping^33,34^, RNA splicing^35^ and protein-protein interactions by fragment complementation^36,37^.

The aim of this work was (i) to develop a novel detection method of Mel using a cell bioasssay based on BLA- assay associated to the human Mel receptor 1B gene (*MTNR1B)* (ii) to compare this bioassay-method with LC-MS/MS, a validated method for Mel detection.

## Materials and Methods

### Chemicals and reagents

Mel (TLC grade, purity ≥98%) was purchased from Sigma-Aldrich (St. Louis, MO, USA). Formic acid was provided by ChemLab (Zedelgem, Belgium), and methanol for liquid chromatography was supplied by J. T. Baker (Fisher Scientific, The Netherlands). Ultrapure water was obtained using a Milli-Q system (Millipore, Milford, MA, USA). Cell culture reagents were purchased from Gibco (Life Technologies, Carlsbad, CA, USA).

Mel standard stock solution was prepared before each use by dissolving a known amount of Mel in absolute methanol (2 g/L). Standard solutions were prepared before each use by diluting Mel standard stock solution in different chemical matrices.

### Sample preparation

Samples with known concentrations (ranging from 0.001 to 100 ng/mL) of Mel in different chemical matrices (synthetic must, white wine and assay medium (AM)) were analysed. Mel was extracted by chloroform^38^ with some modifications. Briefly, 50 µL of sample was mixed with 50 µL of Milli-Q water. Then, 500 µL of chloroform was added and vortexed for 1 min at room temperature. Samples were shaken at 1200 rpm for 1 hour at room temperature. Organic phases from each sample were evaporated until dry under nitrogen gas. The residue was re-dissolved in 50 µL of a methanol/water mixture (40:60, v:v) or in 50 µL of AM (Dulbecco’s-modified Eagle’s medium (DMEM) supplemented with 1% dialyzed Fetal Bovine Serum (FBS), 0.1 mM non-essential amino acids (NEAA), 25 mM HEPES (pH 7.3), 100 U/mL penicillin and 100 µg/mL streptomycin) and centrifuged at 15000 rpm for 5 min at room temperature. Supernatants were analysed using LC-MS/MS or the cell-based bioassay.

### Cell line

Human bone osteosarcoma epithelial cells containing the human Mel receptor 1B (Tango *MTNR1B*-bla U2OS cell line) were obtained from Invitrogen (Carlsbad, CA, USA). The receptor is linked to a TEV protease site, and the Gal4-VP16 transcription factor is stably integrated into the Tango GPCR-bla U2OS parental cell line. Moreover, this parental cell line stably expresses a beta-arrestin/TEV protease fusion protein and the BLA reporter gene under the control of a UAS response element (TANGO^TM^ cell line).

### Cell culture

TANGO cells were maintained in McCoy’s 5 A medium supplemented with 10% dialyzed FBS, 0.1 mM NEAA, 25 mM HEPES (pH 7.3), 1 mM sodium pyruvate, 100 U/mL penicillin, 100 µg/mL streptomycin, 200 µg/mL zeocin, 50 µg/mL hygromycin and 100 µg/mL geneticin. The cells were cultured at 37 °C in an incubator with 5% CO_2_ in 75 cm^3^ cell culture flasks. All experiments were performed with TANGO cells between passages 7 and 11.

### Cell line optimization and analysis

The BLA cell-based bioassay was tested in several conditions: cell number per well (from 1.25 × 10^3^ to 5 × 10^5^), presence or absence of FBS, stimulation time and cell adhesion before analysis to optimize the analysis. After optimization, cells were washed with 10 mL of phosphate-buffered saline (PBS) and dispensed at a concentration of 2 × 10^4^ cells per well on a 96-well black polystyrene plate with flat and clear bottom (Greiner Bio One, Austria) with 40 µL of staining solution, 50 µL of sample and 160 µL of AM. Cells were exposed to different known concentrations of Mel for 24 hours.

Mel determination was carried out according to the LiveBLAzer FRET-B/G Loading Kit (Invitrogen, Carlsbad, CA, USA). Fluorescence intensity was analysed using a POLARstar Omega microplate reader (BMG LABTECH, Germany) with excitation and emission wavelengths of 390 and 450–520 nm, respectively. The results were calculated as a fluorescence ratio (FR) between blue fluorescence, representing the FRET system product as a result of MEL presence, and green fluorescence from the FRET system substrate.

### LC-MS/MS analysis

The Mel concentration was analysed by performing liquid chromatography mass spectrometry following the method described by Rodriguez-Naranjo *et al*.^9^ with some modifications. The system was based on a high-performance liquid chromatograph coupled to a triple quadrupole mass spectrometer (Agilent G6410; Agilent Technologies, Palo Alto, USA). Mel separation was performed using an Agilent Zorbax Sb-Aq column (150 × 2.1 mm i.d., 3.5 µM). Chromatographic separation was performed using a binary gradient consisting of (A) water and (B) methanol as LC grade solvents, both containing 0.1% (v/v) formic acid. The elution profile was 100% B (4 min) and 10% B (6 min). The flow rate was 0.4 mL/min. The injection volume was 7 µL.

Mel quantification was performed using Agilent MassHunter WorkStation Quantitative Analysis software version B0104 by comparing the 233/174 transition MS data of the sample and the standard.

The matrix effect was calculated as the percentage of the matrix-matched calibration slope (X) divided by the standard calibration slope (Y). If the ratio (X/Y × 100) is 100%, there is no matrix effect.

## Results and Discussion

In this study, we aimed to develop and optimize a new method for the detection of Mel in food samples using a BLA cell-based bioassay. For this purpose, a validated method, LC-MS/MS, was used as a reference method to determine whether this methodology could be a good alternative to analyse Mel in food samples.

Therefore, the first step was to implement the LC-MS/MS method developed by Rodriguez-Naranjo *et al*.^9^ to determine the LOD and LOQ in different matrices.

### LC-MS/MS

Matrix-matched calibration curves were prepared with Mel standards (ranging from 100 to 0.05 ng/mL) in different matrices, such as a methanol/water mixture (40:60), Milli-Q water, white wine, grape must and AM. Samples were extracted by chloroform and injected into the LC-MS/MS system. Chloroform is one of the most common solvents used in indolamine extraction procedures, resulting in fewer residues that could affect Mel determination by chromatography^38^. In addition, liquid-liquid extraction is an inexpensive method that can be easily adapted to different types of biological samples^39^.

**Figure 1:**
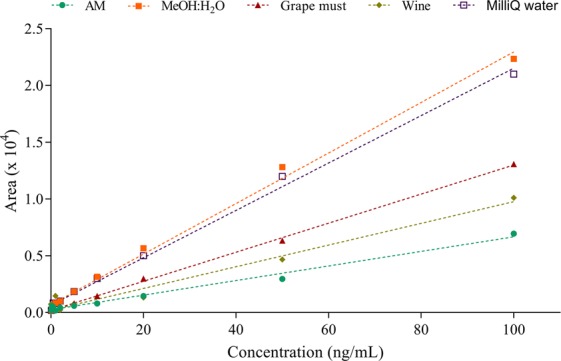
Matrix-matched calibration curves in different media: AM (Assay medium), MeOH:water mixture, milliQ water, grape must and wine. All samples were analyzed by triplicate.

Complex matrices (AM, wine, and grape must) showed a signal suppression of the area response, resulting in a lower percentage of matrix calibration (28.6, 42.0 and 57.1%, respectively) (Fig. 1, Table 1). It is important to notice that even though Mel presented good R^2^ on calibration curves in complex matrices, only high concentrations (from 100 to 5 ng/mL) of Mel were on the linearity of these curves. Limits of detection (LOD) and limits of quantification (LOQ) determination were obtained as 3 and 10 times the signal-to-noise ratio, respectively^40^. LOD (s/n = 3) was 1.03, 0.21 and 0.57 ng/mL, and LOQ (s/n = 10) was 3.10, 0.64 and 1.73 ng/mL for AM, wine and grape must matrices, respectively (Table 1). However, common solvents used for chromatographic analysis, such as a methanol/water mixture (40:60) or Milli-Q water, did not present practically matrix effect, and the LOD and LOQ were very similar (Table 1) due to the lack of primary or secondary metabolites that could interfere with chloroform extraction and chromatographic analysis. The LOD of our method on the determination of Mel was similar to the values reported in previous studies^9,12^ but much higher than the LOD obtained by Fernandez-Cruz *et al*.^17^, with an LOD of 0.0047 ng/mL and an LOQ of 0.0144 ng/mL.

**Table 1:** Calibration curve parameters for Mel determination by LC-MS/MS *vs* BLA cell-based biosensor in different matrices. LOD (limit of detection), LOQ (limit of quantification).

Matrices	LC-MS/MS			Cell biosensor		
	LOD (ng/mL)	LOQ (ng/mL)	Matrix effect (%)	LOD (ng/mL)	LOQ (ng/mL)	EC50 (nM)
MeOH:water	0.17	0.53	100.0	n.d.	n.d.	n.d.
MilliQ water	0.19	0.57	93.3	n.d.	n.d.	n.d.
Grape must	0.57	1.73	57.1	0.21	0.67	0.96
Wine	0.21	0.64	42.0	0.12	0.41	9.51
Assay medium	1.03	3.10	28.6	0.03	0.41	0.06

n.d. not determined.

### Cell-based method optimization

To optimize the BLA cell-based bioassay, several conditions that affect cell growth and detection, such as cell number per well, presence or absence of FBS, stimulation time and cell adhesion before analysis, were first studied in AM with Mel standards (ranging from 100 ng/mL to 0.001 pg/mL) within the range of linearity described by the BLA cell-based bioassay.

**Figure 2:**
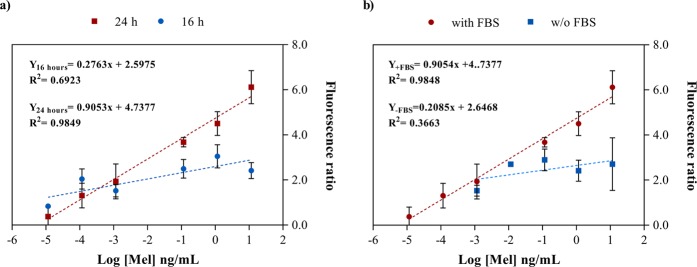
Effect of stimulation time with Mel (**a**) and presence of FBS (**b**) on efficacy of BLA cell-based assay.

Regarding the cell number per well condition, concentrations of 2 × 10^4^ and 5 × 10^4^ cells per well were suitable for Mel determination (with higher FR ratios), with the lowest concentration being the best (Table 2). Higher FR ratios implied that the calibration curve is better and quantification of Mel in samples can be performed. Fewer cells per well were not sufficient to monitor Mel content due to the low fluorescence ratio (FR < 1). Other parameters studied included the presence or absence of FBS, a common supplement to *in vitro* and *ex vivo* cell cultures, and a stimulation time of 16 or 24 hours (Fig. 2). Longer stimulation times with Mel (24 hours) showed a higher signal detection of FR data and improved linearity (R^2^ = 0.9848) (Fig. 2a) than after 16 hours of stimulation. In addition, FBS presence improved FR data (Fig. 2b) due to its rich content of essential components, such as hormones, vitamins, protein transporters, and cell spreading and growth factors^41^. For cell adhesion, we compared the results using adhered cells or cell suspensions at the moment of Mel addition, being better when cells were a suspension (data not shown).

**Table 2:** Effect of cell number on detection by BLA cell-based biosensor.

Cell number per well	Maximum fluorescence ratio (FR)
1,250	<1
2,500	<1
5,000	<1
10,000	<1
20,000	6.1
50,000	5.6

Other fluorescent studies have been performed for Mel determination using enzyme-linked immunosorbent assays (ELISA) in grape skin and beer samples with successful detection results^18,22^. However, according to Rodriguez-Naranjo *et al*.^9^, complex matrices, such as wine or other plant materials, did not provide a linear response by ELISA. Many molecules of the sample may interfere with ELISA determination by structurally mimicking Mel or by cross-reactions within the immunoassay^42^, resulting in false-positives^43^. According to Horwitz^44^, the appearance of either false-negative or false-positive results is typical of trace analysis. Therefore, the specificity of the MTNR-1B receptor used in this method for Mel detection was determined by testing the response of different compounds related to tryptophan and its metabolism (tryptophan, serotonin, and tryptophan ethyl ester, Fig. S2). No signals were detected with any of these compounds; therefore, the receptor presented a high specificity and no cross-reactions.

**Figure 3:**
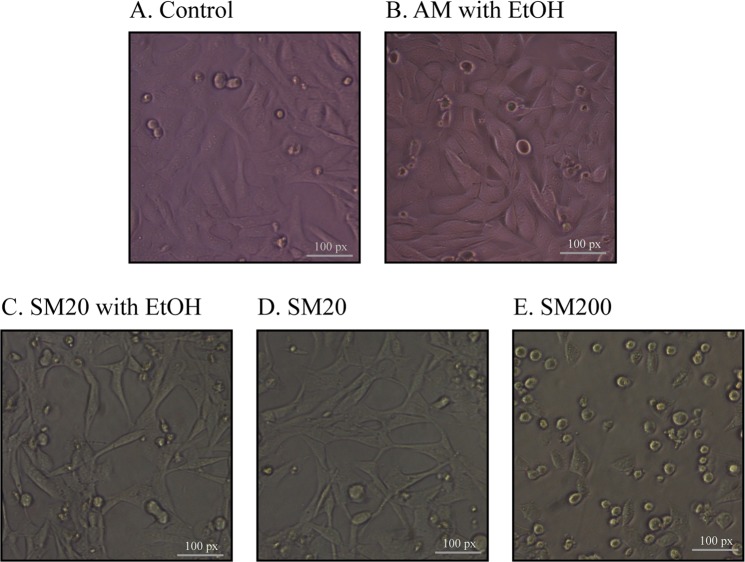
Chemical matrix effect on growth of TANGO cells. (**a**) Control (AM); (**b**) AM with ethanol 12% (v/v); (**c**) Synthetic must (SM) with ethanol 12% (v/v) and 20 g/L of sugar; (**d**) SM with 20 g/L of sugar; (**e**) SM with 200 g/L of sugar.

Food samples, such as wine or must, are complex matrices with different metabolites (ethanol and sugar molecules), which may interfere in BLA cell-based assays because of their effect on cell lines^45^. Therefore, to validate the BLA cell-based bioassay for Mel in food samples, we established the range of linearity in several chemical matrices, such as synthetic must, white wine, Milli-Q water and PBS, by diluting the Mel standard stock solution in the different media. A low response was achieved when complex media were directly applied to the cells (Fig. 3). The presence of sugar and/or ethanol considerably affected cell line growth. Ethanol increased cell volume and compromised cell viability (Fig. 3b); while sugar resulted in decreased pH and cell volume due to osmotic effects (Fig. 3c,d). Erickson *et al*.^46^ demonstrated similar results in chondrocytes; osmotic stress was caused significant cell volume changes. Cell volume decreases as extracellular osmolality increases, resulting in a logarithmic relationship^47^. Nevertheless, in a high concentration of sugar (200 g/L), similar to regular grape must, cells were not able to adhere to the surface (Fig. 3e); thus, mammalian cells lost their viability, and no fluorescence signal was detected.

Therefore, for the determination of Mel in food samples, an extraction step was required for sample preparation to eliminate non-specific interference prior to the detection of Mel. To compare our results with Mel determinations by LC-MS/MS, which was used in this study as reference method, the same extraction protocol with chloroform that is used in this chromatographic method was also applied to these samples.

### Comparison of HPLC vs the cell-based bioassay

A range of several Mel concentrations, from 100 to 0.01 ng/mL, were added to grape must and white wine, extracted by chloroform and resuspended in AM or a methanol/water mixture (40:60), depending on the method used for analysis, the BLA cell-based bioassay or LC-MS/MS, respectively.

**Figure 4:**
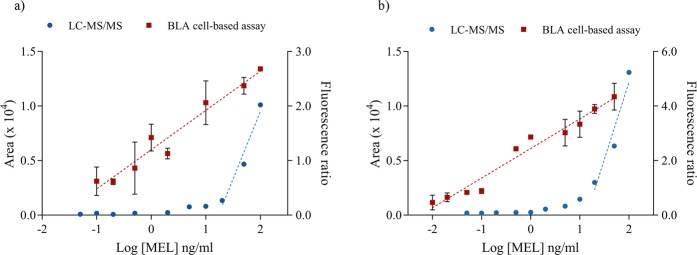
BLA cell-based assay and LC-MS/MS analysis comparison in two different matrices (**a**) wine and (**b**) synthetic must.

Although a signal decrease was observed for both methods (Fig. 4 *vs*. Figures 1 and 2, Table 1), the BLA cell-based bioassay showed increased sensitivity and quantification of Mel in grape must and wine samples (LOD = 0.21 and 0.12 ng/mL and LOQ = 0.67 and 0.41 ng/mL, respectively). Another parameter that provides sensitivity information is the EC50 (refers to the concentration of Mel which induces a half response of the bioassay), which was 0.06 nM in our cell assay with optimized conditions. Nevertheless, the EC50 increased in complex chemical matrices, such as grape must or wine samples (0.96 and 9.51 nM, respectively), due to the presence of primary or secondary metabolites, which could interact with the fluorescent assay system. Kunapuli *et al*.^28^ reported an EC50 of 2.2 nM with a BLA cell-based assay in mammalian cell lines using a cell detection method similar to the BLA cell-based assay proposed in this study. Although in Fig. 1 it was not appreciable because of the scale used, LC-MS/MS analysis showed a loss of linearity at 5 ng/mL of Mel when complex matrices were used (Fig. 4). However, the BLA-cell bioassay maintained a good response even at these low concentrations in complex matrices, resulting in a higher sensitivity (Fig. 4). Consequently, this method allows to quantify Mel at low concentrations.

### Sample analysis

Fermented beverages have been associated with the presence of bioactive compounds, which protect against cardiovascular and neurodegenerative diseases^13^. In particular, melatonin biosynthesis has been shown to be related to aromatic amino acid metabolism in yeast^9,12^. Therefore, to validate our fluorescent BLA cell-based bioassay, we analysed Mel content in 15 samples from alcoholic fermentation and compared with the results obtained by LC-MS/MS (Table 3). During this process, Mel is produced by yeast in small quantities (from pg/mL to ng/mL)^48^. The BLA cell-based bioassay detected Mel in all samples analysed in a range between 0.39 and 23.68 ng/mL. However, Mel was detected only in five samples analysed by LC-MS/MS. Surprisingly, those samples did not coincide with the samples that were determined to have higher concentrations of Mel by the BLA cell-based bioassay. The concentrations of Mel in most samples were very close to the LOD obtained in complex media, such as wine or grape must, which may justify the poor quantification in many of these samples (not quantified). Although the necessity to do a sample pre-treatment previous to the analysis of complex food samples, produced an important reduction of the signal response in both methods. This decrease was lower in the case of the BLA cell-based bioassay, allowing us to detect and quantify the low concentrations of Mel synthesized by yeasts during the alcoholic fermentation. Conversely, in LC-MS/MS analysis, chloroform extraction drastically affected the Mel determination in complex matrices (Fig. 1), reducing the signal and producing the lack of detection in most fermented samples.

**Table 3:** Mel quantification in samples from an alcoholic fermentation by BLA cell-based biosensor *vs* LC-MS/MS.

Sample	Cell biosensor (ng/mL)	LC-MS/MS (ng/mL)
1	0.43	n.q.
2	0.58	n.q.
3	23.68	0.51
4	1.46	n.q.
5	1.39	n.q.
6	0.39	0.84
7	0.68	0.67
8	1.34	0.92
9	0.56	n.q.
10	5.06	n.q.
11	0.59	0.27
12	0.48	n.q.
13	0.99	n.q.
14	0.41	n.q.
15	1.43	n.q.

n.q. non quantified.

## Conclusions

This investigation reports a comparative study on melatonin detection by two different methods, chromatographic analysis and a BLA cell-based bioassay. The study showed an improvement in Mel determination by the BLA cell-based bioassay due to increased sensitivity and improved quantification. Although a noticeable reduction of the signal was observed when Mel was analysed in complex matrices, such as grape must or wine, due to the necessity of sample extraction prior to Mel detection, this bioassay is a good alternative to determine Mel content in food samples.

## Supplementary information

Supplementary Information
