# Supplementary material for: Determination of melatonin by a whole cell bioassay in fermented beverages
Source: Sci Rep. 2019 Jun 24;9:9120. doi: 10.1038/s41598-019-45645-7 (PMC6591416; doi:10.1038/s41598-019-45645-7)

Supplementary information

Determination of melatonin by a whole cell bioassay in fermented beverages

María Ángeles Morcillo-Parra, Gemma Beltran, Albert Mas, María-Jesús Torija*

Departament de Bioquimica i Biotecnologia, Facultat d'Enologia, Universitat Rovira i Virgili, Tarragona, Spain

Figure S1. Overview of BLA system mechanism in absence (A) or in presence (B) of Mel in the medium.

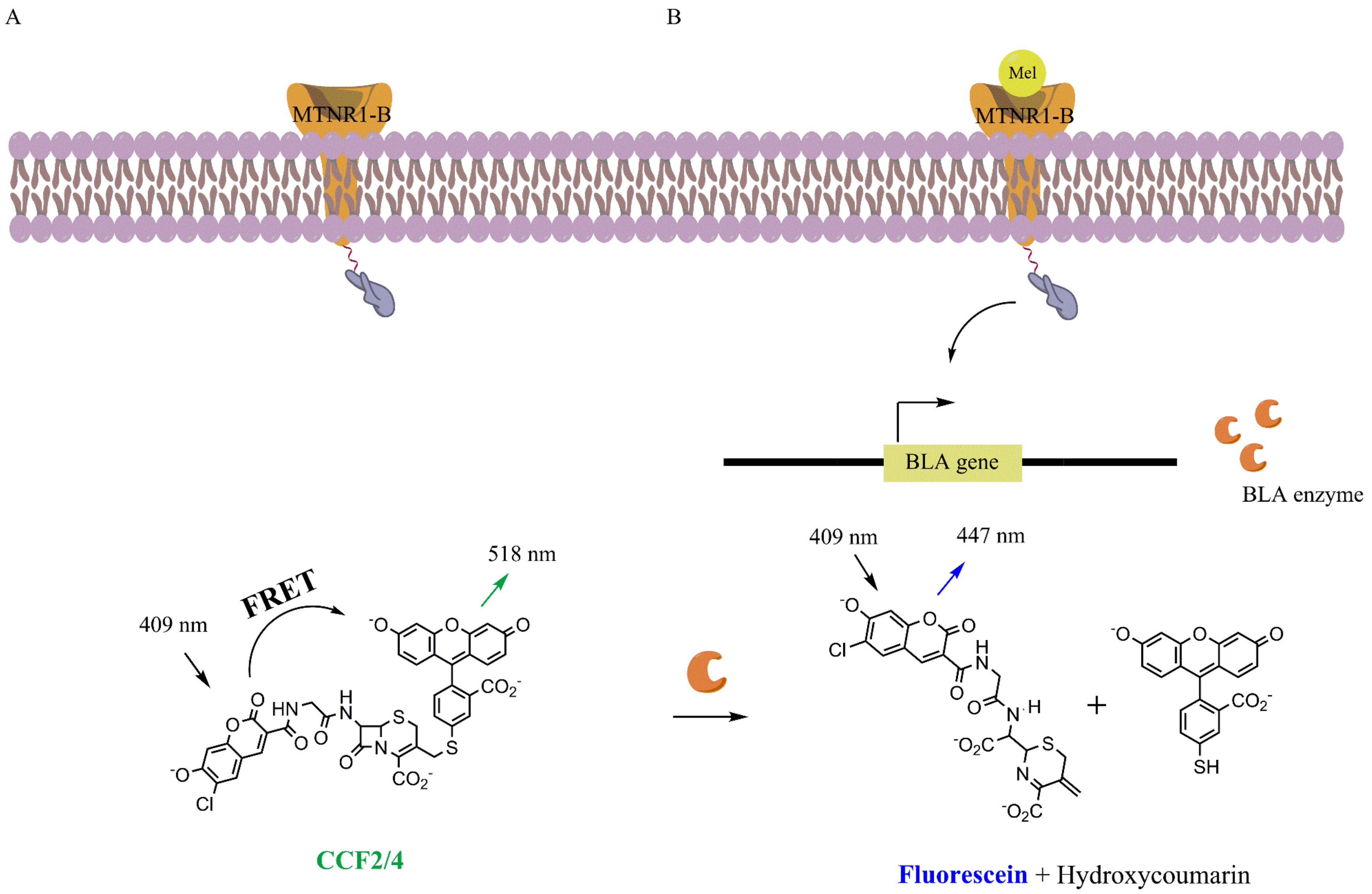

Figure S2. Response of the MTNR-1B receptor to different compounds related to tryptophan and its metabolism.

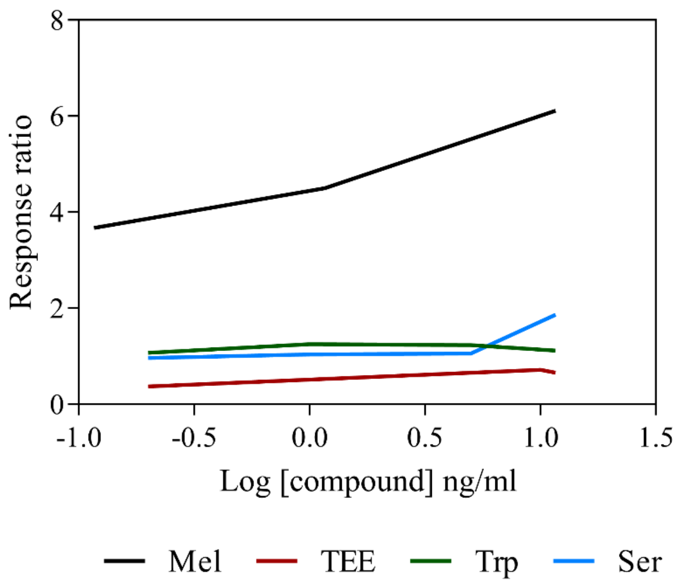

Supplement: Supplementary file 1 — Supplementary Information [file 41598_2019_45645_MOESM1_ESM.pdf]
